# Kinematic Analysis of Bionic Elephant Trunk Robot Based on Flexible Series-Parallel Structure

**DOI:** 10.3390/biomimetics7040228

**Published:** 2022-12-05

**Authors:** Qitao Huang, Peng Wang, Yuhao Wang, Xiaohua Xia, Songjing Li

**Affiliations:** 1 Harbin Institute of Technology, School of Mechatronics Engineering, Harbin 150001, China; 2 Key Laboratory of Road Construction Technology and Equipment of MOE, Chang’an University, Xi’an 710064, China

**Keywords:** flexible rod, cosserat theory, kinematic, flexible series-parallel structure, bionic elephant trunk robot

## Abstract

Researchers borrow ideas from biological characteristics and behavior in design to make bionic robots that can meet unstructured and complex operating environments. The elephant trunk has been widely imitated by bionic robots because of its strong dexterity and stiffness adjustability. Due to the complex structure of the current elephant trunk robot, a series-parallel elephant trunk robot based on flexible rod actuation and a 6-degree-of-freedom (6-dof) parallel module is proposed in this paper. The bionic robot has a simple structure and redundant kinematics, which can realize the control of stiffness. This work focuses on the modeling of the flexible driving rod, the kinematics of a single parallel module, and the whole biomimetic robot. The kinematics are verified by simulation, which lays a foundation for future research on stiffness regulation.

## 1. Introduction

The design inspiration for bionic robots comes from all kinds of creatures in nature, that is, the structural characteristics and behavior of a certain animal or its organs are studied, and the corresponding biomimetic structure is developed [1]. In nearly 60 million years of evolutionary development, the elephant trunk gradually became a versatile organ, strong and powerful, and can achieve many functions and unique advantages such as carrying, grasping, drinking, and attacking. The trunk of an elephant can be bent in the form of a helix, Q, S, U, C, L, I, etc. From the head to the end of the trunk, the size of the trunk decreases, with each segment increasing in rotatable angle and flexibility. And when it comes to different tasks, the stiffness of the trunk can adapt to different environments. The trunk is so versatile that elephants don’t have to adjust their posture to survive like other giant animals. To sum up, the dexterity and stiffness adjustability of the elephant trunk enable it to adapt to various complex environments. Thus, the elephant trunk has become an inspiration and research target of biomimetic robots.

Since the 1960s, researchers have carried out research on bionic series-parallel robots [2]. Victor Scheinman and Larry Leifer of Stanford University built the robot ORM [3]. The ORM includes 28 air bags and seven metal discs. Anderson and Horn have developed a tendon-driven robot that can be used to operate under sea, using plates connected with gimbal joints [4]. Due to the lack of theoretical study for bionic series-parallel robots, the kinematics and dynamics analysis methods of traditional robotic arms are not applicable, which leads to the problems of poor load capacity and low positioning accuracy of the two robots. Walker’s team has made a lot of contributions to the research of bionic series-parallel robots. In 2001, Hannan and Walker et al. developed an elephant-trunk robot with super redundant degrees of freedom [5]. The total length of the elephant-trunk robot is 82.32 cm, including 16 hook joints, each of which has 2 degrees of freedom. In 2002, based on the results of the 2001 research, Walker’s team developed a robot that can bend in a plane [6,7]. The planar series-parallel robot consists of four segments, each of which contains two degrees of freedom. Each section is connected in series by tension structure. The robot can grasp objects in space. In 2005, Walker worked with McMahan on a novel series-parallel robot called Air-Octor [8,9]. Air-Octor uses a pneumatic structure as the support of the robot body, which is bent by three ropes and internal air pressure control. In 2007, Neppalli and Jones combined Air-Octor with OctArm to create a robot with a simple structure and efficient obstacle avoidance ability. The main body of the continuous robot is a rubber tube, and three ropes are distributed uniformly in the circumferential direction of the rubber tube to drive and control it, so that it can achieve the effect of bending in space [10]. In 2004, Nabil Simman and Kai Xu et al. proposed a laryngeal surgical robot based on a series-parallel structure. The robot can bend and rotate, and operate with a tiny metal grasper at the front [11,12,13,14]. In 2007, Choi et al. invented a series-parallel endoscopic robot based on a spring skeleton [15]. The robot is driven by three ropes, with three degrees of freedom, and springs link its internal adjacent joints together, which can achieve bending deformation in three directions. In 2011, Festo developed a new bionic operating arm based on the characteristics of the elephant trunk [16]. Each joint of this bionic robot is composed of airbags, which can smoothly complete the handling of objects and has a strong load capacity. In 2015, Thien-Dang Nguyen et al. proposed elastic magnetic structures with telescopic functions and promoted the degrees of freedom to form stretchable flexible series-parallel robots [17]. Shugen Ma et al. mainly studied the path planning [18] and motion control [19,20] of bionic series-parallel robots. And the design parameters of the robot are optimized based on dynamics [21].

Through this literature, it can be seen that the design of the current bionic series-parallel robots is relatively complex, and its kinematics and dynamics analysis still need to be further studied. Meanwhile, the characteristic of the stiffness adjustability of the elephant trunk has not been studied and reproduced in the scheme of the robot.

**Figure 1:**
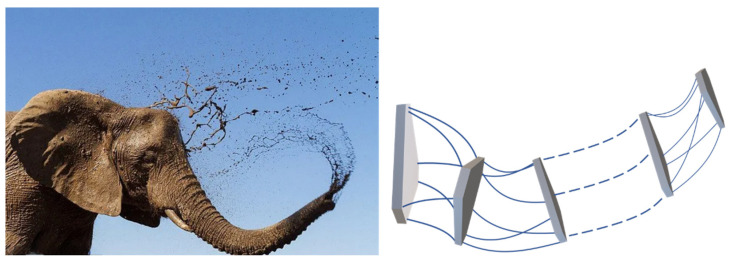
Elephant trunk robot based on flexible series-parallel structure.

In this paper, a series and parallel biomimetic elephant trunk robot based on a flexible rod and a 6-dof parallel module is proposed, as shown in Figure 1. The modeling and kinematics of the driving unit, single parallel module, and series-parallel robot are researched. This paper is organized as follows: Section 2 presents the modeling of the flexible rod which would drive the robot to realize 6-dof motion. In Section 3, kinematic analysis is carried out of the flexible 6-dof parallel manipulator, as the single module of the serial-parallel robot. Finally, the kinematics of the serial-parallel robot are deduced and simulated based on the modeling of flexible rods and the single parallel module.

The flexible rod is chosen as the driving unit on the single module and the whole robot to simplify our design. In a robot system, the driving unit is the core of the system. And it plays an important part in the kinematic analysis. Thus, in this work, our analysis begins with the modeling of a flexible rod as the driving unit of the single parallel module and proposed elephant trunk robot. Note that our work is based on Cosserat theory [22,23,24], and the open-source project Elastica and PyElastica [25,26,27] provide a reliable environment to simulate our model in Python.

## 2. Modelling of Flexible Rod

### 2.1. Geometric Description of The Flexible Rod

The geometric model of the flexible rod contains its geometric and topological information. When the slender flexible rod is subjected to external force, it has large and local deformation, which makes its geometric form complex and diverse, e.g., bending, twisting, and winding. When describing the position of the rod, the geometric shape of a single flexible rod can be expressed by the shape of its center line and the orientation of the cross-section. The shape of the flexible rod’s center line includes bending deformation, and the cross-section could have torsional deformation around the center line. This part describes the geometric parameters of the center line of a single flexible rod and the geometric parameters of the cross-section characteristics, and then describes the spatial geometry of the flexible rod. The expression of the position and shape of flexible rods in 3D space is the prerequisite for the establishment of robot system modeling.

**Figure 2:**
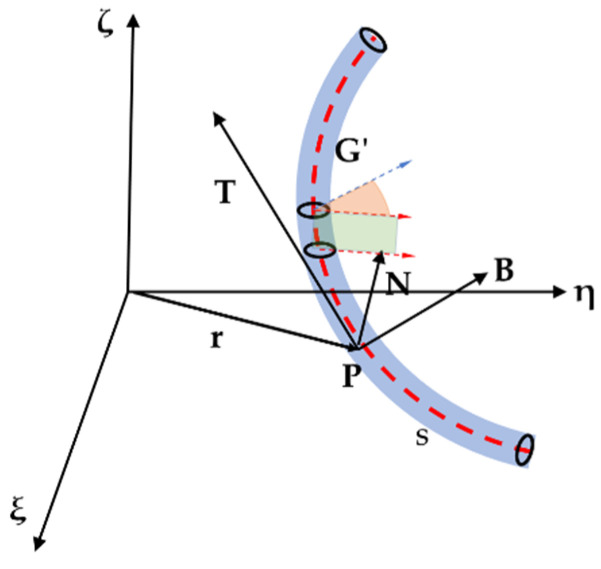
Coordinate systems of the flexible leg.

From the perspective of geometry, the center curve can be regarded as a space curve **G’** with arc length ***L***. The four basic coordinate systems of the legs are established based on the smooth curve **G’**, as shown in Figure 2.

The **P-NBT** coordinate system is called the Frenet coordinate system. The T-axis is the tangent vector of the rod’s center curve **G’** at point **P**, which is defined as Equation (1). The N-axis is the normal vector of the center curve **G’** at point **P**, as defined in Equation (2). Axis B is the binormal vector of **G’** at point **P**, which is defined as Equation (3). The three coordinate axes **N**, **B,** and **T** are pairwise orthogonal. At point **P**, the close plane of the point is determined by the **T** axis and **N** axis. The plane defined by **N** and **B** is called the normal plane.

(1) $$T\left(s\right)=\frac{dr}{ds}$$

(2) $$N\left(s\right)=\frac{1}{\left|T^{\prime }\left(s\right)\right|}\frac{dT}{ds}$$

(3) B(s)=T(s)×N(s)

**Figure 3:**
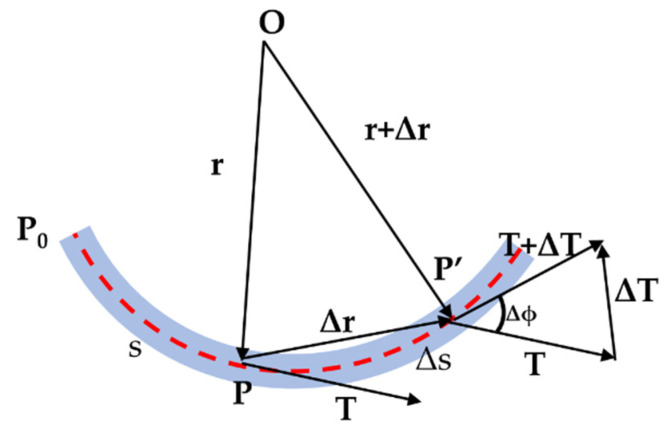
Differential geometry of a space curve.

The curvature and torsion of the curve can completely determine its spatial orientation [22]. Consider an arc of length ds between two points **P** and **P’** on the space curve **G’**, as shown in Figure 3, Let dϕ be the angle subtended by ds at the center of a circle with radius **r**. Since ds=rdϕ, the change of the tangent vector with *ds* is approximately the same with the change of dϕ. As a result, the curvature **κ** of a space curve is defined by Equation (4).

(4) $$\kappa \left(s\right)=\underset{\Delta s\to 0}{\lim}\left|\frac{\Delta \phi }{\Delta s}\right|=\underset{\Delta s\to 0}{\lim}\left|\frac{\Delta T}{\Delta s}\right|=\left|\frac{dT}{ds}\right|=\frac{d\phi }{ds}$$

(5) $$\tau \left(s\right)=\left|\frac{dB}{ds}\right|$$

**τ** is the torsion of the curve which could be calculated by Equation (5). While the curvature **κ** is a measure of the deviation of the curve from a straight line, the torsion **τ** is a measure of the twisting of the curve out of the osculating plane.

The Darboux vector $\omega_{F}$ is defined as Equation (6), whose physical meaning is the angular rotation velocity of the Frenet coordinate system (**p-NBT**) relative to the inertial coordinate system (**o-ξηζ**) when point **P** moves along the center curve **G’** in the positive direction of the arc coordinate **s** with unit velocity. The variation of vectors **N**, **B** and **T** can be determined by Darboux vector $\omega_{F}$.

(6) $$\omega_{F}=\kappa \left(s\right)B+\tau \left(s\right)T$$

Given curvature ***κ*(*s*)** and torsion ***τ*(*s*)**, the variation of each coordinate axis vector of Frenet coordinate system (**p-NBT**) with arc coordinates **s** can be solved according to Equation (7), and then the shape of the curve can be obtained by Equation (1), as shown in Equation (8). Curvature ***κ*** and torsion **τ** are two independent variables to determine the shape of a space curve, so the degree-of-freedom of space curve is 2.

(7) $$\{\begin{array}{l} \frac{dN}{ds}=\omega_{F}\times N=\tau \left(s\right)B-\kappa \left(s\right)T\\ \frac{dB}{ds}=\omega_{F}\times B=-\tau \left(s\right)N\\ \frac{dT}{ds}=\omega_{F}\times T=\kappa \left(s\right)N \end{array}$$

(8) $$r\left(s\right)=\int_{0}^{s}T\left(\sigma \right)d\sigma +r\left(0\right)$$

After determining the center curve, the orientation of the cross-section of the rod is further determined to obtain the spatial shape of the rod. The rod is jointly determined by the orientation of its center curve and cross-section. After the center curve of the rod is determined, only the torsion angle of the cross-section needs to be obtained, as shown in Figure 2. Therefore, each rod has 3 degrees of freedom.

ω is defined as the angular velocity related to the torsional deformation of the rod, as shown in Equation (9). Its physical meaning is the angular velocity of the cross-section rotation relative to the inertial coordinate system (**o-ξηζ**) when the point **P** on the center curve G ‘of the rod moves in the forward direction with unit velocity.

(9) $$\omega =\omega_{F}+\left(\frac{d\chi }{ds}\right)z$$

where, χ is the angular and $\left(\frac{d\chi }{ds}\right)z$ is the relative angular velocity of the section with respect to the Frenet coordinate system (**P-NBT**), and z is the tangent of the center curve. $\omega_{F}$ is the relative angular velocity of the coordinate system (**P-NBT**) with respect to (**O-ξηζ**). The projected component of the angular velocity ω on the axis of the coordinate system (**P-XYZ**) on the cross-section is shown in Equation (10).

(10) $$\omega_{x}=\kappa \sin\chi ,\omega_{y}=\kappa cos\chi ,\omega_{z}=\tau +\frac{d\chi }{ds}$$

Thus, the shape of the rod can be expressed by three independent variables $\omega_{x}$, $\omega_{y}$ and $\omega_{z}$.

Similar to Equation (7). The variation of the coordinate system (**P-XYZ**) with the arc coordinate **s** can be described by the ω(s), as shown in Equation (11).

(11) $$\left(\frac{dx}{ds},\frac{dy}{ds},\frac{dz}{ds}\right)=\left(\omega \times x,\omega \times y,\omega \times z\right)$$

### 2.2. Static Equilibrium of Flexible Rod

**Figure 4:**
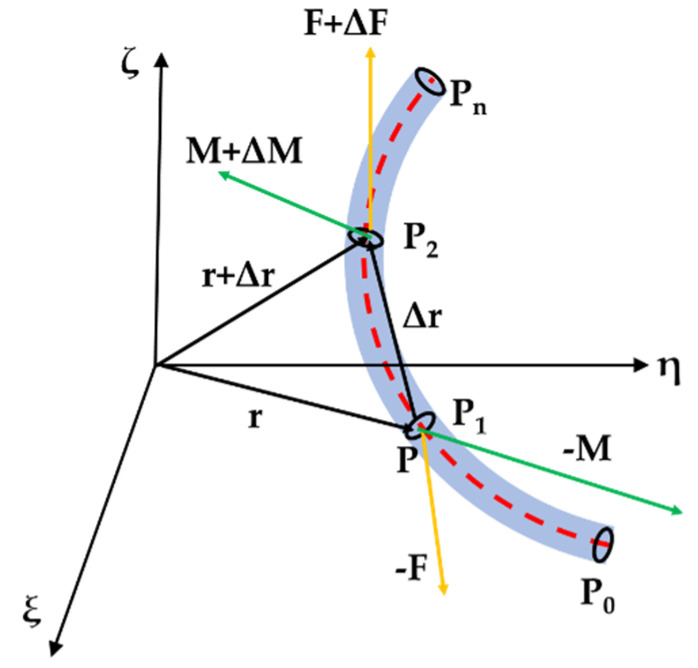
Static equilibrium of rod.

For the flexible rod, it is divided into micro segments, and each micro segment is analyzed by equilibrium analysis. As shown in Figure 4, **P1** and **P2** are 2 infinitely closed points on the rod. The reference position vector of origin point O of the inertial coordinate system are **r** and **r + Δr**, respectively, and their relative arc coordinates are **s** and **s + Δs**, respectively. The static equilibrium is established by taking the micro-element of the rod as the unit, and the internal forces and internal moments of the external section of **P1** point are set as ***-F*** and ***-M****,* respectively, and the internal forces and internal moments of the external section of **P2** point are F+ΔF and M+ΔM, respectively. In the equilibrium state at P1, the resultant force and moment of force or torque are 0, as shown in Equation (12).

(12) $$\{\begin{array}{l} \Delta F=0\\ \Delta M+\Delta r\times F=0 \end{array}$$

Taking the derivative of Equation (12) to arc coordinates **s** and converting it to the coordinate system (**P-XYZ**):

(13) $$\{\begin{array}{l} \frac{dF}{ds}+\omega \times F=0\\ \frac{dM}{ds}+\omega \times M+z\times F=0 \end{array}$$

When there is no original curvature, the moment of micro segment on the rod can be expressed through ω as:

(14) $$M_{x}=A\omega_{x},M_{y}=B\omega_{y},M_{z}=C\left(\omega_{z}-\omega_{z}^{0}\right)$$

where *A* and *B* are the flexural stiffness of the rod section around the *X*-axis and *Y*-axis, respectively, *C* is the torsional stiffness of the rod section around the *Z*-axis. They could be obtained:

(15) $$A=EI_{x},\;B=EI_{y},\;C=GI_{Z}$$

In Equation (15),

$I_{x}$: The moment of inertia of the cross-section with respect to the *x* axis, $I_{x}=\pi d^{4}/64$.

$I_{y}$: The moment of inertia of the cross-section with respect to the *y* axis, $I_{y}=\pi d^{4}/64$.

$I_{z}$: The moment of inertia of the cross-section with respect to the z axis, $I_{z}=\pi d^{4}/32$.

*E*: Young’s modulus.

*G*: Shear modulus G = E/(2 + 2μ).

μ: Poisson ratio

*d*: Diameter of cross section of rod

Equation (12) projected to the coordinate system (**P-XYZ**) can be transformed into:

(16) $$\{\begin{array}{c} \frac{dF_{x}}{ds}+\omega_{y}F_{z}-\omega_{z}F_{y}=0\\ \frac{dF_{y}}{ds}+\omega_{z}F_{x}-\omega_{x}F_{z}=0\\ \frac{dF_{z}}{ds}+\omega_{x}F_{y}-\omega_{y}F_{x}=0 \end{array}$$

(17) $$\{\begin{array}{l} A\frac{d\omega_{x}}{ds}+\left(C-B\right)\omega_{y}\omega_{z}-C\omega_{z}^{0}\omega_{y}-F_{y}=0\\ B\frac{d\omega_{x}}{ds}+\left(A-C\right)\omega_{z}\omega_{x}+C\omega_{z}^{0}\omega_{x}+F_{x}=0\\ C\frac{d\omega_{z}}{ds}+\left(B-A\right)\omega_{x}\omega_{y}=0 \end{array}$$

Equations (16) and (17) are static balance equations of the flexible rod. It contains six variables *F_x_*, *F_y_*, *F_z_* and *ω_x_*, *ω_y_*, *ω_z_*. Given the initial condition of the ordinary differential equations, the shape of the rod can be obtained. After the variation of *F_x_*, *F_y_*, *F_z_* and *ω_x_*, *ω_y_*, *ω_z_* with arc coordinate **s** are obtained, the variation of curvature **κ**, torsion **τ**, and torsion Angle χ with arc coordinates **s** could be solved by Equation (10). By Equation (11), it is transformed into the variation of the axis of the coordinate system (**P-XYZ**) with arc coordinate **s**.

## 3. Modelling of Single Flexible 6-Dof Parallel Module

**Figure 5:**
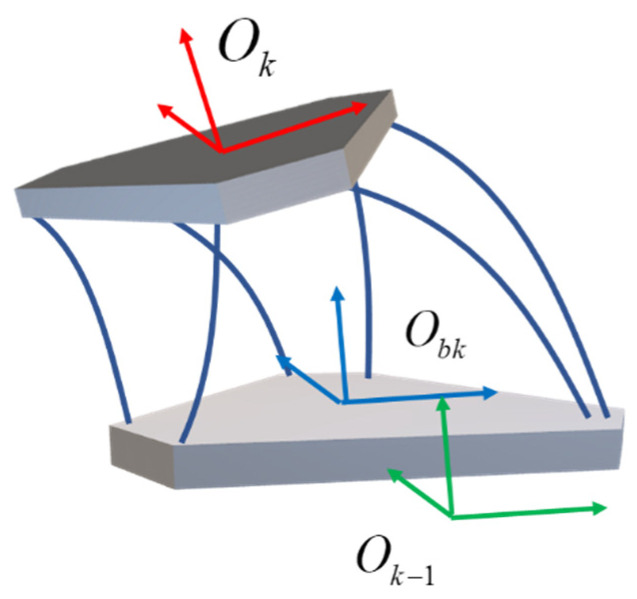
Prototype Design of k-th Flexible Parallel Module.

One flexible parallel module has six driven flexible rods and has a similar structure to the rigid 6-dof Stewart platform, as shown in Figure 5. The flexible rods are clamped and fixed on the moving platform. The flexible rods pass through the base platform and can translate through the holes on the base platform and can be braked so that these six flexible rods can be driven independently.

Based on the basis of the previous sections, we modeled the flexible rod based on Cosserat theory. In the following sections, the forward and inverse kinematics and the static equilibrium of a single parallel module are introduced.

The shape of each flexible rod consisting of the module is determined by the position function $P_{i}\left(s_{i}\right)\in {\mathbb{R}}^{3}$ and the orientation function $R_{i}\left(s_{i}\right)\in \mathrm{SO}\left(3\right)$ of arc length $s_{i}\in \mathbb{R}$, constituting a reference system $g_{i}\left(s_{i}\right)=\left[\begin{array}{cc} R_{i}\left(s_{i}\right) & P_{i}\left(s_{i}\right)\\ 0 & 1 \end{array}\right]\in \mathrm{SE}\left(3\right)$ attached to the flexible rod. The position and attitude of the flexible rod evolve with the kinematic parameters $v_{i}\left(s\right)\in {\mathbb{R}}^{3}$ and $u_{i}\left(s\right)\in {\mathbb{R}}^{3}$ of arc length s, which represent the linear velocity and angular velocity of the rod, respectively.

(18) $$\begin{array}{l} P_{i}{}^{\prime }=R_{i}v_{i}\\ {R_{i}}^{\prime }=R_{i}{\hat{u}}_{i} \end{array}$$

where, ′ denotes the differential of $s_{i}$, ^ denotes the mapping from ${\mathbb{R}}^{3}$ to SO(3) (antisymmetric matrix). Similarly, use the ∨ representing inverse mapping (that is, the mapping from SO(3) to ${\mathbb{R}}^{3}$).

The variation of internal force $F_{n}$ and moment $M_{n}$ with arc length $s_{i}$ can be described by the classical Cosserat rod differential equations of static equilibrium:

(19) $$\begin{array}{l} F_{ni}{}^{\prime }=-F_{i}\\ M_{ni}{}^{\prime }=-{r_{i}}^{\prime }\times F_{ni}-M_{i} \end{array}$$

The kinematic variables $v_{i}$ and $u_{i}$ are related to material strains (shear, elongation, bending, and torsion) for the calculation of internal force and moment.

(20) $$\begin{array}{l} F_{ni}=R_{i}K_{se,i}\left(v_{i}-v_{i}^{*}\right),K_{se,i}=\left[\begin{array}{ccc} A_{i}G_{i} & 0 & 0\\ 0 & A_{i}G_{i} & 0\\ 0 & 0 & A_{i}E_{i} \end{array}\right]\\ M_{ni}=R_{i}K_{bt,i}\left(u_{i}-u_{i}^{*}\right),K_{bt,i}=\left[\begin{array}{ccc} E_{i}I_{i} & 0 & 0\\ 0 & E_{i}I_{i} & 0\\ 0 & 0 & J_{i}G_{i} \end{array}\right] \end{array}$$

where, $v_{i}^{*}$ and $u_{i}^{*}$ are the kinematic parameters of the rod without external force and moment. For the flexible rod whose initial state is straight, the two parameters are $v_{i}^{*}={\left[\begin{array}{ccc} 0 & 0 & 1 \end{array}\right]}^{T}$ and $u_{i}^{*}={\left[\begin{array}{ccc} 0 & 0 & 0 \end{array}\right]}^{T}$, respectively. The matrix $\;K_{se,i}$ and $K_{bt,i}$ are the stiffness terms of the radial symmetric cross-section rod, which varies with the arc length, including cross-sectional area $A_{i}$, Young’s modulus $E_{i}$, shear modulus $G_{i}$, moment of inertia of section $I_{i}$ (about **PX** and **PY** axes), polar area moment $J_{i}$ about **PZ** axis. Therefore, for each flexible rod, Equations (18)–(20) constitute a system of differential equations, which describes the evolution of the variable $P_{i}$, $R_{i}$, $F_{ni}$ and $M_{mi}$ with arc length $s_{i}$.

### 3.1. The Boundary Conditions for Forward Kinematics

Each flexible rod in the robot is independently described by the above differential equations. However, the boundary conditions of each set of differential equations are coupled due to the physical constraints in the robot structure.

For the 6-dof flexible parallel module configuration proposed in the previous. The proximal end of each flexible rod is clamped in a groove connected to the moving platform with a fixing screw. And each rod passes through a cylindrical hole in the base platform. This configuration constrains the intersecting position $P_{i}$ of the bar and the base platform and the tangent vector of the rod at this point, while allowing the rod to rotate around the tangent. Therefore, there is no torque around the tangent direction. $R_{i}\left(0\right)$ is expressed as the orientation when rotating $\theta_{i}$ around the *z*-axis of the global coordinate system, then:

(21) $$\begin{array}{l} M_{niz}\left(0\right)=0\\ R_{i}\left(0\right)=\left[\begin{array}{ccc} \cos\theta_{i} & -\sin\theta_{i} & 0\\ \sin\theta_{i} & \cos\theta_{i} & 0\\ 0 & 0 & 1 \end{array}\right] \end{array}$$

At the end of each rod ($s_{i}=L_{i}$) interacting with the moving platform, the following static equilibrium conditions must be satisfied:

(22) $$\begin{array}{l} \sum_{i=1}^{n}\left[F_{ni}\left(L_{i}\right)\right]-F=0\\ \sum_{i=1}^{n}\left[p_{i}\left(L_{i}\right)\times F_{ni}\left(L_{i}\right)+M_{ni}\left(L_{i}\right)\right]-p_{c}\times F-M=0 \end{array}$$

where, F and M, respectively, represent the external force and moment acting on the point $p_{c}$ of the moving platform. According to the same shape of each rod at $s_{i}=L_{i}$, the constrain about $R_{i}$:

(23) $$R_{i}\left(L_{i}\right)=R_{1}\left(L_{1}\right)$$

The following equation can then be written about the position of the end of each rod:

(24) $$p_{1}\left(L_{1}\right)-p_{i}\left(L_{i}\right)-R_{1}\left(L_{1}\right)\left(a_{1}-a_{i}\right)=0\;\mathrm{for}\;i=2\cdots n$$

where, $a_{i}$ is the position of the joint linking the i-th rod and moving platform in the moving platform coordinate system.

$p_{1}$ and $R_{1}$ are unknown to be solved. Thus, there are 12 unknowns in forward kinematics. Equation (22) has six sub-equations that could be regarded as the main equations. And Equations (22) and (24) consist of the equations of constraint, which have six sub-equations. Therefore, after solving these 12 equations together, the position $p_{1}$ and orientation $R_{1}$ of the moving platform could be obtained.

### 3.2. The Boundary Conditions for Inverse Kinematics

For the inverse kinematic, the boundary conditions given in Equations (21) and (22) are still reasonable. However, the geometric coupling of the flexible rods on the moving platform is simplified because the position and orientation of the moving platform are known. The orientation of each rod’s end ($R_{i}\left(L_{i}\right)$) is consistent with the orientation of the moving platform ($R_{d}$):

(25) $$R_{i}\left(L_{i}\right)=R_{d}$$

Similar to Equation (24), the joints’ position on the moving platform could be expressed as the corresponding rod’s end position ($p_{i}$) and relative position of the center of the moving platform ($p_{d}$).

(26) $$p_{i}=p_{d}+R_{d}a_{i}\;\mathrm{for}\;i=1\cdots n$$

In the inverse kinematic, $R_{d}$ and $p_{d}$ should be given as the input command. 

In the inverse kinematic, there are six unknowns ($L_{i}$) to be solved. Similar to the forward kinematic, each length of the rod could be contained by solving Equations (22) (25) and (26).

We note that Equations (23) and (25) are multi-dimensional with more than nine unknows in one equation. It would be easier if R(SO(3)) could be simplified to ${\mathbb{R}}^{3}$. Thus, in the actual solution process of kinematics, Equations (23) and (25) are always transferred to:

(27) $${\left[\log\left(R_{i}^{T}\left(L_{i}\right)R_{1}\left(L_{1}\right)\right)\right]}^{\vee }=0\;\mathrm{for}\;i=2\cdots n$$

(28) $${\left[\log\left(R_{i}^{T}\left(L_{i}\right)R_{d}\right)\right]}^{\vee }=0\;\mathrm{for}\;i=1\cdots n$$

log() takes the natural logarithm of the matrix which could map SO(3) to so(3). And ∨ maps so(3) to ${\mathbb{R}}^{3}$.

### 3.3. Smulation of Single Flexible 6-Dof Parallel Module

**Figure 6:**
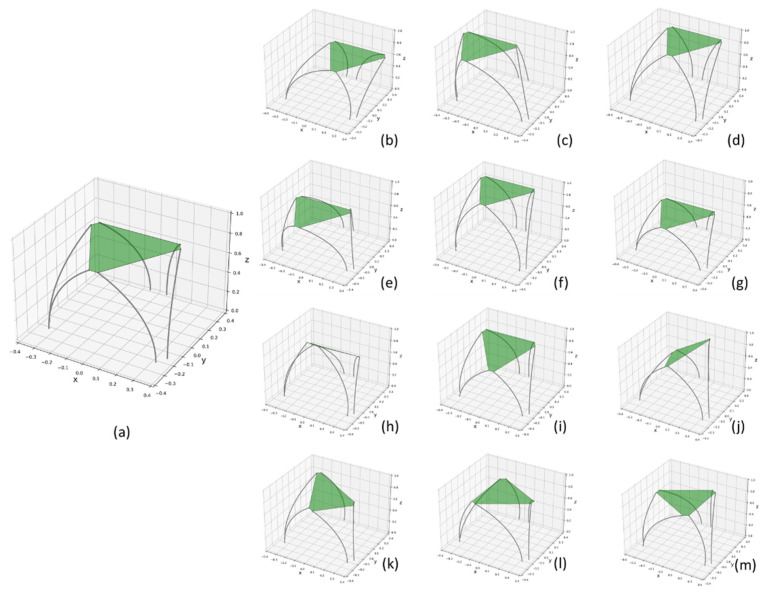
A 6-dof simulation for one flexible module. (**a**): initial orientation, (**b**,**c**): translation ±200 mm along *x*-axis, (**d**,**e**): translation ±200 mm along *y*-axis, (**f**,**g**): translation ±200 mm along *z*-axis, (**h**,**i**): rotation ±45° across *x*-axis, (**j**,k) rotation ±45° across *y*-axis, (**l**,**m**) rotation ±30° across *z*-axis.

Taking the rigid steward platform as a reference, here the flexible parallel module is simulated on 6-dof motion, including translation and rotation by the *x*, *y*, and *z* axis. From Figure 6, the single flexible parallel module could realize 6-dof movements. And none of the rod has interference among these motions. This could be regarded as the basis of imitation of a trunk’s behavior. Meanwhile, the result could support the design and simulation of the whole flexible series-parallel robot.

## 4. Kinematic Modeling of Flexible Series-Parallel Mechanism

According to different types of series and parallel manipulators, their kinematic modeling processes can be divided into the following three types:

(1) **Continuum Serial-Parallel Manipulator**. A continuous series-parallel robot is composed of several parallel elements but does not contain discrete joints and rigid rods, so its shape is usually characterized by a curve. For the kinematic modeling of this kind of series-parallel robot, the shape curve should be described in space first, and then the pose of each unit on the curve should be determined according to the structural characteristics, and then the kinematic parameters of the mechanism should be derived.
(2) **Discrete Serial-Parallel Manipulator having less than or equal to 6-dof**. For discrete series-parallel robots, if the joint degrees of freedom are less than or equal to 6, the transformation matrix relative to the root coordinate system or the world coordinate system can be derived according to the position and orientation of the end-effector. Then, the kinematic parameters can be determined according to the structure and geometric constraints of the robot. For the intermediate platforms, it is not necessary to know their specific position and orientation before the solution kinematic. Because of the series and parallel robots with degrees of freedom ≤6, once the position and orientation of the end-effector are determined, each intermediate platform can be uniquely determined. In other words, the transformation matrix of the end-effector contains the kinematic information of each intermediate platform, but these parameters need to be derived by using the structural and geometric constraints of the robot.
(3) **Discrete Serial-Parallel Manipulator having greater than 6-dof.** For discrete series-parallel robots, if the joint degrees of freedom are greater than six, it means that the robot has redundant degrees of freedom, and the displacement of each joint cannot be completely determined according to the orientation of the end-effector. Therefore, for such discrete series-parallel robots, the inverse kinematics should be solved by giving or solving the orientation of intermediate platforms first. The forward kinematics also need to determine each intermediate platform in turn.

**Figure 7:**
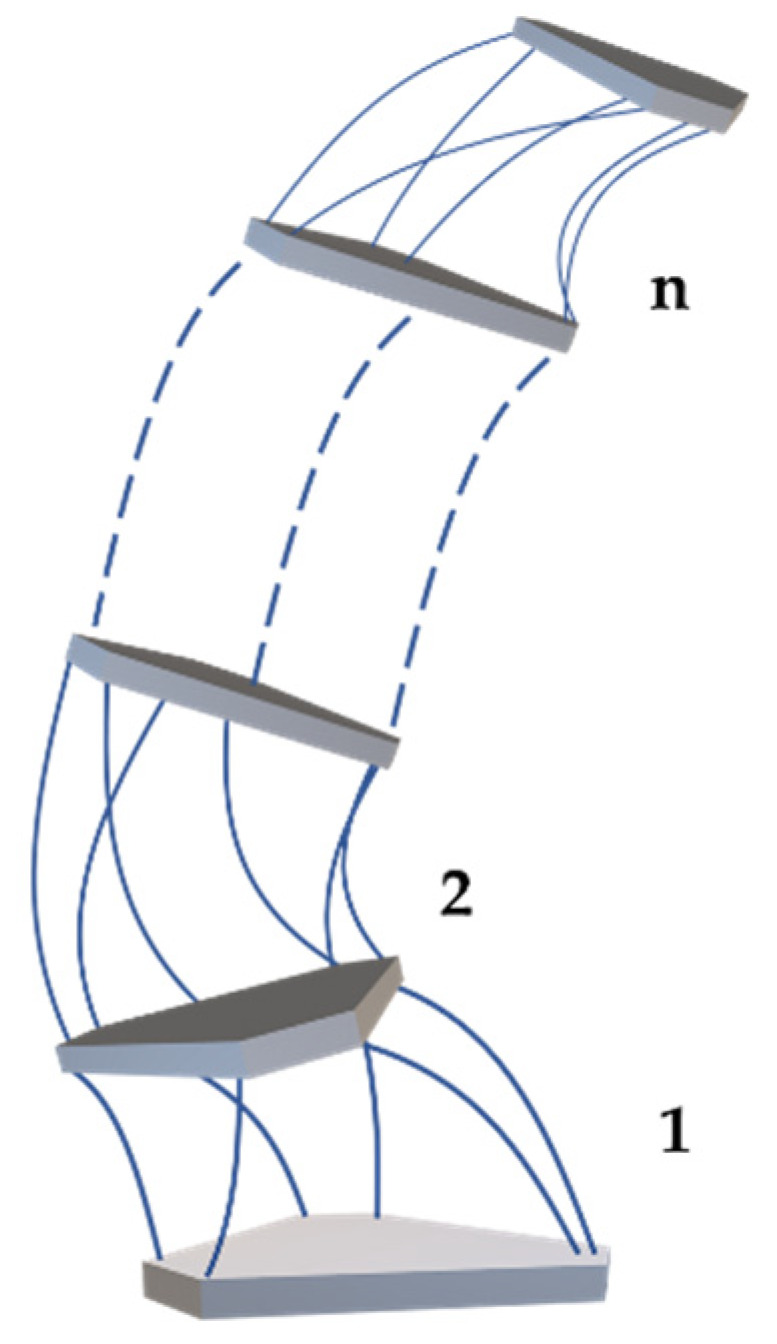
Series-parallel robot and n parallel modules.

A series-parallel robot consists of *n* parallel modules connected in series to a fixed base as shown in Figure 7. For the sake of explanation, assume that these parallel modules are similar in structure. Figure 5 depicts the structure of a single module. The degree of freedom of its moving platform relative to the base of the module is $N_{k}$. And it has $m_{k}$ flexible rods for driving. Define $\sum_{k}$ and $\sum_{bk}$ are coordinate systems fixed on the module k’s moving and base platform, respectively. Coordinate system $\sum_{0}$ is the base of module 1. Since the base of the module k is fixed on the moving platform of the module k−1, the transformation matrix between $\sum_{k-1}$ and $\sum_{bk}$ is a constant matrix, namely: [Rk−1bkPbk,k−101]=cons.

### 4.1. The Kinematic Model of The Module K

The forward and inverse kinematic expressions of the module are given as follows:

(29) $$\left[\begin{array}{cc} R_{k} & P_{k}\\ 0 & 1 \end{array}\right]=F\left(L_{k}\right)\;k=1,\ldots ,n$$

(30) $$L_{k}=f\left(\left[\begin{array}{cc} R_{k} & P_{k}\\ 0 & 1 \end{array}\right]\right)\;k=1,\ldots ,n$$

where, $R_{k}$ and $P_{k}$ are the orientation matrix and position vector of the moving platform of the module k. $L_{k}$ is the vector composed of the joint displacement of each flexible rod in module k. F() is the mapping from the joints’ displacement and the moving platform’s position and orientation. f() is the inverse mapping from the moving platform’s position and orientation and the joints’ displacement. Because the elastic behavior of large deflection is involved, and the analytical expression cannot be obtained in most cases, it needs to be obtained by the numerical calculation method, as discussed in Section 3.1 and Section 3.2.

### 4.2. Forward Kinematics Analysis of Flexible Series-Parallel Robot

The forward kinematics of a flexible series-parallel robot is to obtain the position and orientation of each intermediate platform and end effector by knowing the displacement of each active joint. The orientation of the moving platform of each module in the global coordinate system (${R^{\prime }}_{k}$ and ${P^{\prime }}_{k}$) can be deduced by the orientation of its moving platform with respect to its base platform ($R_{k}$ and $P_{k}$) using the recursive formula (k=1,…,n):

(31) R′k=Rk⋅Rk−1bk⋅R′k−1  k=2…nP′k=P′k−1+RkPk    k=2…nR′1=R1P′k=P1

$R_{k}$ and $P_{k}$ in the equation can be obtained according to Equation (29). ${P^{\prime }}_{0}$ is the position vector of the base platform of module 1. When k=n, ${R^{\prime }}_{n}$ and ${P^{\prime }}_{n}$ are the orientation of the end-effector (end-moving platform). 

**Figure 8:**
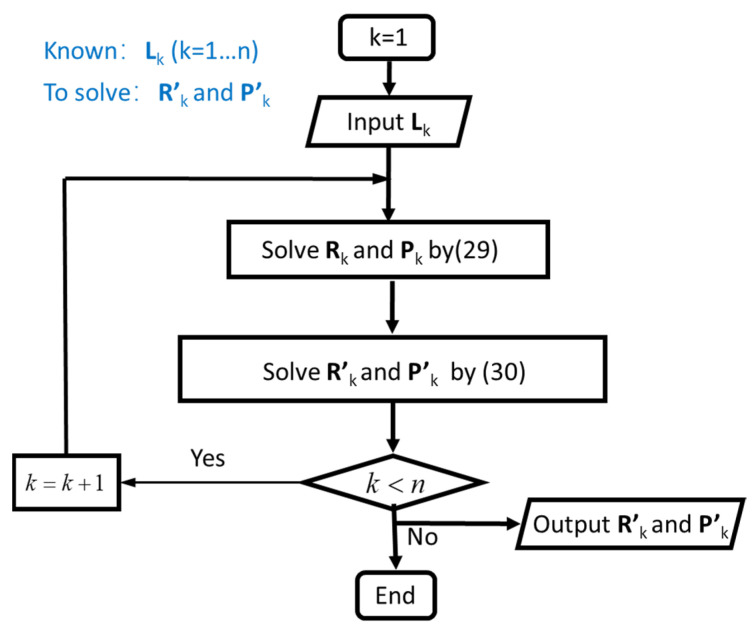
Flow chart of forward kinematics solution.

At first, by the input $L_{1}$, the orientation and position of 1-th moving platform with respect to its base platform ($R_{1}$ and $P_{1}$) could be obtained by Equation (29). Then, the orientation and position of 1-th moving platform with respect to the global coordinate system (${R^{\prime }}_{1}$ and ${P^{\prime }}_{1}$) could be obtained by Equation (30). Note that, when k = 1, $R_{1}$ and $P_{1}$ is identical to ${R^{\prime }}_{1}$ and ${P^{\prime }}_{1}$. After that, the arithmetic iterates once by k + 1. Repeat the solution of $R_{k}$ and $P_{k}$ until k = n. In summary, the forward kinematics solution of the flexible series-parallel robot can be expressed as in Figure 8.

### 4.3. Inverse Kinematics Analysis of Flexible Series-Parallel Robot

The inverse kinematics is a process of solving the displacements of active joints of each module according to the orientation of each intermediate platform. Since the object of study in this paper is a series-parallel robot with degrees of freedom > 6, for the solution of inverse kinematics, the orientation of each platform located in the middle and end needs to be known before. Usually, the orientation of the intermediate platform can be directly provided or obtained according to the end effector combined with the constraint conditions. Since the total degree of freedom of the robot is > 6, there would be multiple solutions to obtain each intermediate platform’s orientation only through the kinematics information of the end effector. Thus, the unique solution is often determined cooperatively with the geometry constraints and parameters to be optimized. The optimized objects are generally obstacle avoidance, time optimization, energy consumption optimization, smoothness optimization, singularity avoidance optimization, and base disturbance optimization. This is named Configuration Planning. For Configuration Planning of series-parallel robots with degrees of freedom > 6, this is equivalent to the attitude planning of soft continuous robots and has been maturely researched. The most basic strategy of Configuration Planning is based on the differential geometry of curves. In this kind of strategy, the basic geometric characteristics of robot orientation are obtained by defining the backbone curve. In order to solve the redundancy problem, the intrinsic geometric function of the backbone curve is restricted to the modal form. This method is directly applied to the manipulator with continuous morphology and can be extended to the Configuration Planning of series-parallel robots with multiple modules. Therefore, in this work, we research the kinematics of a series-parallel robot under a well-planned configuration instead of focusing on Configuration Planning.

Similar to the forward kinematic, the displacement of the active joint of each module also needs to be deduced by a recursive formula. According to Equation (30), the following can be obtained:

(32) Rk=R′k⋅R′k−1T⋅Rk−1bkTk=2…nPk=RkT(P′k−P′k−1)k=2…nR1=R′1k=1P1=P′1k=1

Combined with Equation (29), the active joints’ position of each module can be obtained as follows:

(33) Lk={f([R′1P′01])k=1f([R′k⋅R′k−1T⋅Rk−1bkTRkT(P′k−P′k−1)01])k=2…n

**Figure 9:**
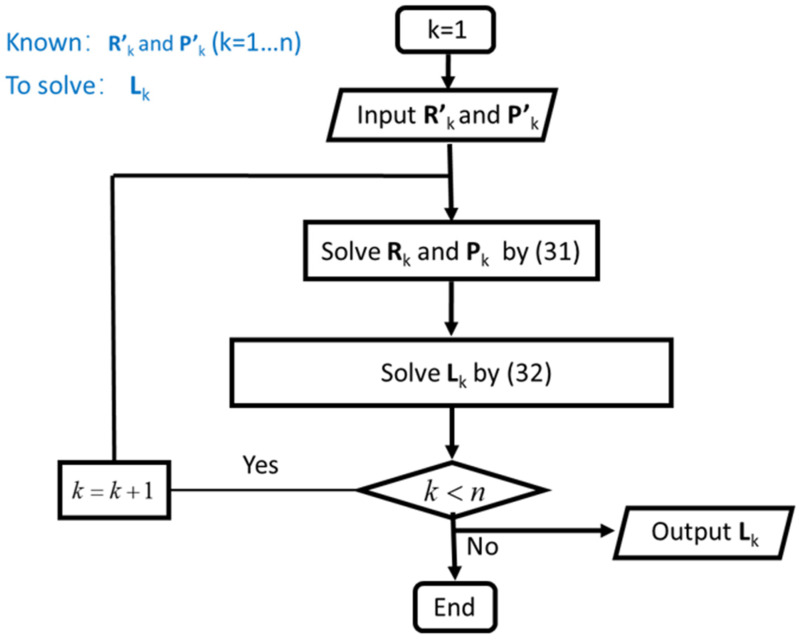
Flow chart of inverse kinematics solution.

To obtain each group of $L_{k}$, a iterative algorithm is proposed such as forward kinematics. At first, input the known orientation and position of 1-th end-effector with respect to the global coordinate system ${R^{\prime }}_{1}$ and ${P^{\prime }}_{1}$. By Equation (31), the orientation and position of 1-th end-effector with respect to its base platform $R_{1}$ and $P_{1}$ could be obtained. Then, $L_{1}$ could be calculated by Equation (32). Make k + 1 and repeat the solution of $R_{k}$, $P_{k}$ and $L_{k}$. Iterate the arithmetic by k + 1 until k = n. In summary, the inverse kinematics solution of the flexible series-parallel robot can be expressed as in Figure 9.

### 4.4. Simulation

**Figure 10:**
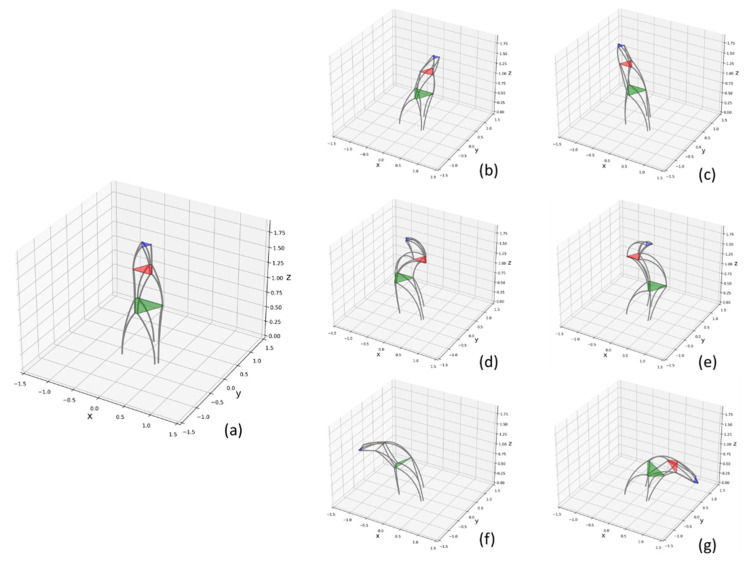
Simulation of flexible series-parallel mechanism. (**a**): initial orientation, (**b**,**c**): translation, (**d**,**e**): s-shaped orientation, (**f**,**g**): bending.

In this section, three parallel modules are connected to constitute a trunk robot (*n* = 3) to verify the previous analysis. According to the trunk’s behavior, a group of motions were simulated, including translation and bending. As shown in Figure 10b,c, the robot could translate in the space. It is helpful to use the robot for carrying. To have a close imitation of an elephant’s trunk, an s-shaped orientation is tested in this section, as shown in Figure 10d,e. The s-shaped orientation is a signature behavior of elephants. It could demonstrate the flexibility of the robot. In Figure 10d,e, the three-module robot can complete the s-shaped orientation roughly. In addition, it can be deduced that the s-shaped orientation would be imitated better if it has more sections. The flexibility is enhanced with increased parallel units. Figure 10f,g show the bending ability of the robot which is a common action when an elephant is grasping and eating. From the result, it can be seen the robot could bend in a specific direction with a relatively small curvature and large angle.

## 5. Conclusions

In this work, an elephant trunk robot based on a flexible series-parallel structure is proposed. The flexible rod as a driven unit is modeled by the Cosserat theory. A single flexible parallel module is analyzed in kinematics. Finally, the whole robot’s kinematics are modeled in the iteration method and the simulation shows the effectiveness of the analysis and its flexibility.

To sum up, we could conclude that:

(1) A flexible rod works well in a flexible series-parallel structure as verified in simulation. In addition, Cosserat theory is an effective method in kinematic analysis of a flexible series-parallel structure and a bionic elephant trunk robot.
(2) Finish the kinematic analysis of a flexible parallel module which makes it a reliable section to consist of a trunk robot.
(3) The trunk robot proposed in this work could closely imitate an elephant’s behavior, such as translating, presenting s-shaped orientation, and bending.
(4) To get better flexibility, more modules should be connected.

## 6. Future Work

The dynamic and stiffness performance would be analyzed based on this kinematics research. Structure parameters need to be optimized to meet the requirements of the stiffness regulating range.
